# MMFNet: A multi-branch multi-scale framework with adaptive sparse self-attention and cross-modal fusion for sleep stage assessment

**DOI:** 10.1371/journal.pone.0353930

**Published:** 2026-07-17

**Authors:** Yuan Li, Ningning Wang

**Affiliations:** School of Physics and Electronic-Electrical Engineering, ABA Teachers College, Aba Tibetan and Qiang Autonomous Prefecture, Sichuan, China; Institute of Wenzhou, Zhejiang University, CHINA

## Abstract

Accurate sleep stage classification serves as a crucial foundation for sleep health assessment and disease diagnosis. However, existing approaches still encounter several challenges, including limited feature representation, redundancy in extracted information, and difficulties in effectively integrating cross-modal physiological signals. To address these issues, we propose a novel framework entitled Multi-Branch Multi-Scale Fusion with Adaptive Sparse Self-Attention and Cross-Modal Integration (MFFNet). Specifically, considering the prominent time-frequency characteristics of sleep signals, multiple branches are designed to capture multi-scale information, including a time-granular feature extraction branch and a time-frequency extraction branch. Furthermore, a multi-head adaptive sparse self-attention mechanism is introduced to suppress redundant information while emphasizing discriminative features. In addition, we employ an adaptive cross-modal fusion strategy that dynamically integrates information from EEG and EOG, and further visualize the contribution of each modality to sleep stage classification. Experiments conducted on the Sleep-EDF-39 and Sleep-EDF-153 datasets demonstrate the effectiveness of the proposed approach. Using the Fpz-Cz EEG channel and EOG signals, MFFNet achieves accuracy rates of 84.26% and 81.86%, with F1 scores of 75.91% and 73.38%, respectively, highlighting its competitive performance in sleep stage assessment.

## Introduction

Accurate sleep stage detection is essential for evaluating sleep quality and constitutes a cornerstone for the clinical diagnosis of sleep-related disorders. Polysomnography (PSG), the gold standard for sleep studies, records multiple physiological signals such as electroencephalography (EEG) and electrooculography (EOG) to assess sleep states [1]. These recordings are typically segmented into 30-second epochs and manually annotated following the guidelines of Rechtschaffen and Kales (R&K) [2] or the American Academy of Sleep Medicine (AASM) [3]. However, this manual scoring process is not only labor-intensive and time-consuming but also prone to inter-rater variability and human error. Consequently, there is a pressing need for automated sleep stage detection methods to reduce the burden of manual annotation while improving efficiency and reliability.

In recent years, the advancement of automatic sleep stage classification has offered a more efficient approach for evaluating sleep quality and diagnosing related disorders. Early studies predominantly employed traditional machine learning methods, which relied on handcrafted features derived from physiological signals. Representative approaches include multilayer perceptrons [4], support vector machines [5], hidden Markov models, Gaussian mixture models [6], and cluster-based adaptive methods [7]. However, the effectiveness of these models largely depends on the quality of the extracted features, which are typically designed based on researchers’ expertise and prior knowledge, thus limiting both performance and generalization ability. For instance, techniques such as the adjustable Q wavelet transform [8] and discrete wavelet transform [5] have been utilized to decompose EEG signals and extract statistical descriptors for subsequent classification. While these methods achieve reasonable performance, they remain heavily dependent on complex feature engineering and domain-specific knowledge, restricting their scalability and robustness.

In contrast, deep learning-based approaches adopt an end-to-end paradigm that enables models to automatically learn discriminative representations directly from raw physiological signals, without relying on handcrafted feature engineering or extensive domain-specific expertise. Commonly used architectures include Convolutional Neural Networks (CNNs) [9], Recurrent Neural Networks (RNNs) [10], Deep Belief Networks (DBNs) [11], and autoencoders [12]. These methods have demonstrated strong capability in modeling the complex and hierarchical patterns of sleep signals, leading to substantial progress in automatic sleep stage classification. For example, Supratak et al. proposed DeepSleepNet [13], which combines CNNs and bidirectional recurrent layers to jointly learn temporal and contextual information from raw single-channel EEG, achieving competitive performance in sleep staging. In addition, SM IN et al. [14] developed a multimodal deep learning model based on EEG, EOG, and CNN-GRU, highlighting the value of integrating complementary physiological modalities for sleep stage classification. Chambon et al. [15] designed a multimodal time-series CNN with embedded spatial filters to improve feature extraction efficiency. Eldele et al. [16] introduced a multi-resolution CNN with attention mechanisms to recalibrate single-channel EEG features adaptively, while Khalili et al. [17] demonstrated that CNNs alone can effectively capture temporal characteristics for reliable sleep stage prediction. Jadhav et al. [18] further transformed EEG signals into time–frequency spectrograms using continuous wavelet transform and employed pretrained CNNs for feature extraction, illustrating the flexibility of deep learning in handling time–frequency representations. Collectively, these studies indicate that multimodal learning, temporal modeling, and multi-scale representation are all promising directions for improving sleep stage classification.

Nevertheless, several important challenges remain. Different sleep stages are characterized by distinct EEG frequency patterns—for example, sleep spindles and K-complexes are prominent in N2, whereas slow-wave activity dominates N3—while EOG signals mainly contain low-frequency components and provide complementary information, especially in stages such as N1 and REM. Although previous studies have explored deep learning, multimodal modeling, and multi-scale feature extraction, effectively capturing stage-specific time–frequency characteristics, suppressing redundant interactions, and adaptively integrating cross-modal information remain open problems. Motivated by these observations, we propose MMFNet, which differs from previous approaches in three main aspects: it employs a physiologically guided multi-branch multi-scale design for EEG and EOG, introduces a Multi-head Adaptive Sparse Self-Attention (MASSA) block to reduce redundancy while preserving essential temporal dependencies, and incorporates an adaptive cross-modal fusion module to dynamically integrate complementary information from different modalities. It is worth emphasizing that, unlike transform-based methods which first convert physiological signals into spectrogram-like or fused image representations before applying two-dimensional neural networks, our approach directly learns complementary temporal and frequency-aware representations from raw one-dimensional signals via physiologically motivated multi-scale convolutions.

**Fig 1:**
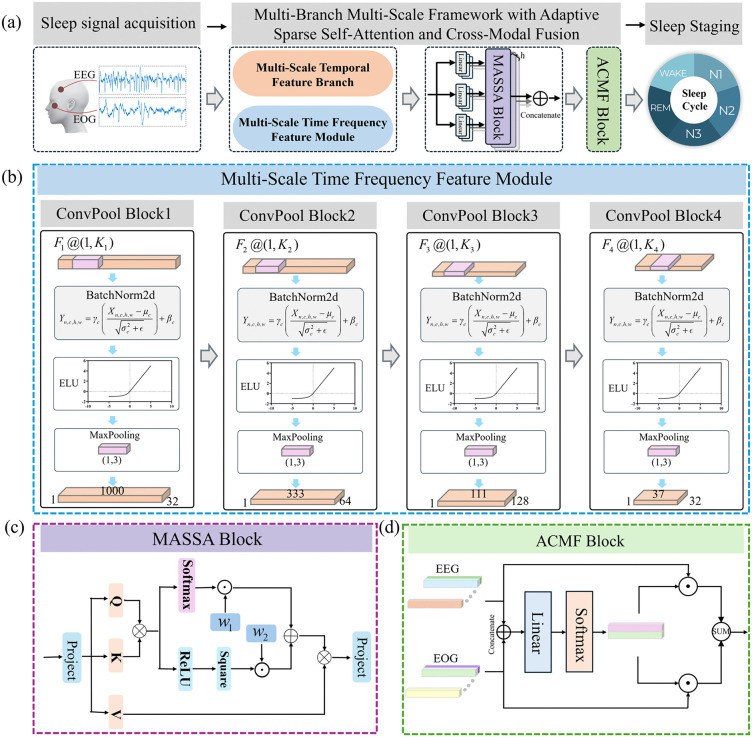
The overall framework diagram of the proposed FM-ASANet in this study.

To tackle the aforementioned challenges, we propose a novel architecture termed MMFNet (illustrated in Fig 1a), which leverages the time–frequency characteristics of physiological signals for automatic sleep stage classification. For EEG signals, three branches are specifically designed at different scales to capture time–frequency features across multiple frequency bands. During downsampling, the convolution kernel size is synchronously adjusted to align the receptive field with the temporal dimension of the input, ensuring that convolution is consistently performed at the appropriate scale of the signal. This design enables more robust extraction of scale-specific time–frequency representations. Considering that EOG signals primarily contain low-frequency components below 10 Hz, two branches are constructed at different scales to capture their distinctive time–frequency patterns. The features learned from different branches are then concatenated along the temporal dimension and processed by a self-attention mechanism to model temporal dependencies. However, direct application of standard self-attention often introduces redundancy and noise due to numerous irrelevant interactions. To address this, we incorporate a sparse branch into the multi-head self-attention mechanism, which filters out low query–key matching scores to suppress adverse effects from irrelevant features, while a dense branch is retained to ensure sufficient information flow for discriminative representation learning. Subsequently, high-level abstract features from EEG and EOG are adaptively fused, integrating the complementary advantages of different modalities to construct richer and more comprehensive representations, thereby improving sleep stage classification performance.

Overall, the main contributions of this study are summarized as follows:

1. Multi-Branch Multi-Scale Time–Frequency Feature Extraction: We design specialized multi-branch, multi-scale feature extraction blocks to capture the time–frequency characteristics of EEG and EOG signals during sleep. Specifically, three branches are constructed for EEG and two branches for EOG to extract diverse high-level features. By synchronously aligning temporal downsampling with convolution kernel lengths, the framework ensures consistent receptive fields across scales, thereby enabling comprehensive extraction of scale-specific time–frequency representations.
2. Multi-head Adaptive Sparse Self-Attention (MASSA) Block for Redundancy Reduction and Efficient Fusion: To address information redundancy in multi-scale feature fusion and noise introduced by conventional self-attention, we propose a Multi-head Adaptive Sparse Self-Attention (MASSA) block. Specifically, the MASSA block contains two complementary attention branches. The first is a sparse self-attention branch, which suppresses weak or irrelevant temporal interactions by filtering out low query–key matching scores, thereby reducing redundancy and noise. The second is a dense self-attention branch, which preserves sufficient information flow to prevent excessive sparsity and loss of useful contextual information. The outputs of these two branches are then adaptively weighted and combined, allowing the model to capture informative temporal dependencies while maintaining robust feature representations.
3. Adaptive Cross-Modal Fusion Block: To exploit the complementary nature of EEG and EOG, we introduce an adaptive cross-modal fusion block. Instead of treating the two modalities equally, this module learns their relative importance and dynamically assigns larger weights to the more informative modality at different time steps. In this way, it enables more effective multimodal integration and generates richer and more discriminative feature representations for sleep stage classification.

## Materials and methods

### Overview of the MMFNet model

In this study, MMFNet comprises: 1). a multi-branch, multi-scale time-frequency feature convolution module, 2). a multi-head adaptive sparse self-attention module, and 3). an adaptive cross-modal information fusion module. MMFNet is an end-to-end model that adaptively extracts temporal and time-frequency features from raw EEG and EOG data and performs accurate classification. The overall structure of the model is illustrated in Fig 1a.

First, we employ multi-scale time–frequency feature extraction blocks to process 30-second EEG and EOG epochs, using three branches for EEG and two branches for EOG. For EEG, convolutional kernels of different sizes are applied to capture features across multiple frequency bands, while temporal downsampling and kernel sizes are adjusted synchronously to maintain scale consistency. Second, we propose a Multi-head Adaptive Sparse Self-Attention (MASSA) module to model temporal dependencies from the high-level time–frequency features extracted by the multi-branch structure. More specifically, this module contains two complementary branches: a sparse attention branch, which suppresses weak or irrelevant temporal interactions by filtering low query–key matching scores, and a dense attention branch, which preserves sufficient contextual information to avoid excessive sparsity. By adaptively combining these two branches, the model can reduce redundancy and noise while retaining informative temporal dependencies. Third, we introduce an adaptive cross-modal fusion module to exploit the complementary characteristics of EEG and EOG. Instead of directly concatenating features from the two modalities, the module first estimates their relative importance at different time steps and then uses these weights to adaptively enhance the more informative modality, thereby producing richer and more discriminative fused representations for sleep stage classification.

To improve reproducibility, we further summarize the detailed architecture of MMFNet in Table 1, including the input size, output size, and convolutional configuration of each module. For both Sleep-EDF-39 and Sleep-EDF-153, the EEG and EOG recordings used in this study were sampled at 100 Hz. Since each sample corresponds to a 30-second epoch, the input size for each modality is (B, 1, 3000), where B denotes the batch size. The table reports the module-wise dimensional changes across the five branches, including the outputs of each ConvPool block, the input/output sizes of the MASSA block, the output size of the ACFM module, and the final classification layer. In this way, the complete feature extraction and fusion process can be reproduced more directly.

**Table 1:** Detailed architectural specifications of MMFNet.

	Block	ConvPool 1			ConvPool 2		ConvPool 3		ConvPool 4		MASSA Block		ACFM	CLS
		IS	OS	Filter	OS	Filter	OS	Filter	OS	Filter	IS	OS	OS	OS
**EEG**	**B1**	(B,1,3000)	(B,32,1000)	32@(1,5)	(B,64,333)	64@(1,5)	(B,128,111)	32@(1,5)	(B,32,37)	32@(1,5)	(B,32,111)	(B,32,32)	(B,32,32)	(B,5)
	**B2**	(B,1,3000)	(B,32,1000)	32@(1,200)	(B,64,333)	64@(1,66)	(B, 128,111)	128@(1,22)	(B,32,37)	32@(1,7)				
	**B3**	(B,1,3000)	(B,32,1000)	32@(1,50)	(B,64,333)	64@(1,16)	(B, 128,111)	32@(1,5)	(B,32,37)	32@(1,1)				
**EOG**	**B4**	(B,1,3000)	(B,32,1000)	32@(1,5)	(B,64,333)	64@(1,5)	(B, 128,111)	32@(1,5)	(B,32,37)	32@(1,5)	(B,32,74)	(B,32,32)		
	**B5**	(B,1,3000)	(B,32,1000)	32@(1,100)	(B,64,333)	64@(1,33)	(B, 128,111)	32@(1,11)	(B,32,37)	32@(1,3)				

Note: *IS* denotes input size, *OS* denotes output size, and Filter denotes the convolutional configuration in each ConvPool block, represented as *F@(1, K)*, where *F* is the number of output channels and *(1, K)* is the convolution kernel size. B1, B2, and B3 denote the three EEG branches, corresponding to the Fine-Grained Temporal Feature Extraction Branch, the Low-Frequency Time–Frequency Branch, and the Mid-Frequency Time–Frequency Branch, respectively. B4 and B5 denote the two EOG branches, corresponding to the Fine-Grained Temporal Feature Extraction Branch and the Mid-Frequency Time–Frequency Branch, respectively.

### Multi-branch multi-scale time-frequency feature extraction block

According to the AASM guidelines, different sleep stages are characterized by distinct waveform patterns in physiological signals. For example, the wake stage is dominated by alpha and beta activity (8–30 Hz), the N1 stage primarily exhibits theta rhythms, the N2 stage is marked by the presence of sleep spindles and K-complexes (8–16 Hz), the N3 stage is characterized by slow-wave activity (0.5–4 Hz), and the REM stage is mainly associated with theta and alpha frequencies. Motivated by the stage-specific spectral characteristics of sleep signals, we introduce a multi-branch, multi-scale feature extraction module to capture complementary information from raw EEG and EOG signals. It should be noted that we do not explicitly transform the input signals into time–frequency representations using methods such as the Fourier transform or wavelet transform. Instead, the proposed branches operate directly on the original one-dimensional signals but use different convolutional kernel lengths and receptive fields to model distinct temporal and frequency-related patterns. Specifically, the fine-grained temporal branch focuses on local temporal dynamics and high-frequency details through short kernels, whereas the time–frequency branches use larger, physiologically designed kernels to capture oscillatory patterns at different temporal scales and frequency ranges. In this sense, the term “time–frequency branch” refers to frequency-aware feature extraction from raw signals through multi-scale convolutions, rather than an explicit transform-based time–frequency image representation. MMFNet incorporates three branches for EEG and two branches for EOG (see Fig 1a), each implemented using four convolutional and max-pooling layers (ConvPool blocks, see Fig 1b).

(1) Fine-Grained Temporal Feature Extraction Branch (FGEFE Branch): In the first branch, convolution is applied along the temporal dimension using small convolutional kernels of size (1, 5). This branch is designed to capture local temporal dynamics, abrupt waveform changes, and relatively high-frequency components in EEG signals. Therefore, it mainly emphasizes short-range temporal structure in the raw signal. To stabilize training, batch normalization is employed to mitigate internal covariate shift, while the ELU activation function is adopted to alleviate the vanishing gradient problem during backpropagation. Furthermore, max pooling is utilized to preserve the most salient features within each segment, and dropout is applied during training to randomly deactivate neurons with a given probability. Together, these strategies enhance the robustness of the learned features and reduce the risk of overfitting.

In this branch, all four ConvPool blocks use convolution kernels of size (1, 5), but the number of kernels varies, specifically 32, 64, 128, and 32, respectively. The max pooling size is set to (1, 3). Let $Y_{temporal}^{i}\in R^{N\times k_{i}\times 1\times f_{i}}$ denote the output of the i-th ConvPool block, N represent the number of samples, $k_{i}$ indicate the number of filters, and $f_{i}$ denote the temporal dimension of the output from the i-th ConvPool block. Given an input $X\in R^{N\times 1\times 1\times 3000}$, the output $Y_{temporal}^{i}$ is obtained as follows:


$$\begin{array}{c} \begin{array}{c} Y_{temporal}^{i}=MP\left(\Phi \left(F_{bn}\left(Conv\left(Y_{temporal}^{i-1},s_{temporal},padding\right)\right)\right)\right)\;\;\\ \;\;\;\;\;\;\;\;\;\;\;\;\;\;(i=1,2,3,4) \end{array} \end{array}$$ (1)

Where $Y_{temporal}^{\text{0}}=X$, $Conv\left(Y_{temporal}^{i-1},s_{temporal},padding\right)$ performs a two-dimensional convolution operation with a kernel size of (1,$s_{temporal}=5$). Padding is applied to ensure that the dimensions remain unchanged before and after convolution.$F_{bn}(\cdot )$ denotes the batch normalization operation, Φ(·) represents the ELU activation function, and MP(·) refers to the max pooling operation with a size of (1,3).

(2) Low-Frequency Time Frequency Branch (LFTF Branch): Unlike the fine-grained temporal branch, this branch is designed to capture longer-duration oscillatory patterns associated with lower-frequency sleep rhythms. The first convolutional layer uses a kernel size of (1, 200). By setting the kernel length to twice the sampling rate, the branch becomes sensitive to activity around 0.5 Hz and above [19]. Importantly, this branch does not generate an explicit time-frequency map; instead, it extracts frequency-related characteristics directly from the raw signal through large convolutional receptive fields. The kernel length $S_{low}^{i}$ of the convolution in the i-th ConvPool block is defined as follows.


$$\begin{array}{c} S_{low}^{i}=\left(1,\left\lfloor \frac{2\cdot F_{s}}{{\text{3}}^{i-1}}\right\rfloor \right),\text{\textbackslash{}hspace\{0.33em}\;\;\;\}i\in \left[1,2,3,4\right] \end{array}$$ (2)

Where, $F_{s}$ is the sampling rate of the signal, and i denotes the i-th ConvPool block, whose convolutional structure is consistent with that of the temporal feature branch. $Y_{low}^{i}\in R^{N\times k_{i}\times 1\times f_{i}}$, $k_{i}$ and $f_{i}$ represent the output of the i-th ConvPool block, the number of filters, and the time dimension after convolution, respectively. It is defined as:


$$\begin{array}{c} Y_{low}^{i}=MP\left(\Phi \left(F_{bn}\left(Conv\left(Y_{low}^{i-1},S_{low}^{i},padding\right)\right)\right)\right)\;\;(i=1,2,3,4) \end{array}$$ (3)

Where $Y_{low}^{\text{0}}=X$.

(3) Mid-Frequency Time–Frequency Branch (MFTF Branch): This branch shares the same structure as the low-frequency branch but uses a smaller kernel size to focus on mid-frequency oscillatory patterns. The first convolutional layer employs a kernel size of (1, 50), corresponding to a frequency resolution of approximately 2 Hz [20]. Similar to the low-frequency branch, this design performs frequency-aware convolution on raw signals, rather than using a separate signal transform such as FFT or wavelet decomposition. The kernel lengths $S_{mid}^{i}$ of the four ConvPool blocks are as follows:


$$\begin{array}{c} S_{mid}^{i}=\left(1,\left\lfloor \frac{F_{s}}{2\cdot {\text{3}}^{i-1}}\right\rfloor \right),\text{\textbackslash{}hspace\{0.33em}\;\;\;\}i\in \left[1,2,3,4\right] \end{array}$$ (4)

Where $Y_{mid}^{i}$ is defined as:


$$\begin{array}{c} Y_{mid}^{i}=MP\left(\Phi \left(F_{bn}\left(Conv\left(Y_{mid}^{i-1},S_{mid}^{i},padding\right)\right)\right)\right)\;(i=1,2,3,4) \end{array}$$ (5)

Where $Y_{mid}^{\text{0}}=X$.

Each branch’s output features from the final ConvPool block are concatenated along the temporal dimension. Therefore, the final output of the three modules, $Y_{all}=R^{N\times k_{\text{4}}\times 1\times \sum f_{\text{4}}}$, is defined as:


$$\begin{array}{c} Y_{all}=\left[Y_{temporal}^{\text{4}},Y_{low}^{\text{4}},Y_{mid}^{\text{4}}\right] \end{array}$$ (6)

Here, [⋅] denotes the concatenation operation along the temporal dimension.

To avoid excessive computational overhead, we designed only two branches for EOG multi-scale time-frequency feature extraction, unlike EEG (Fig 1a). The fine-grained temporal feature extraction branch for EOG follows the same design as that of EEG. Considering that EOG activity is primarily concentrated below 10 Hz, we further introduced a single time-frequency branch that targets frequencies above 1 Hz, employing four convolutional kernel sizes—(1, 100), (1, 33), (1, 11), and (1, 3)—to capture multi-scale patterns.

### Multi-head adaptive sparse self-attention

Self-attention mechanisms have achieved remarkable success in fields such as computer vision and natural language processing [21, 22]. However, conventional self-attention considers all tokens within the feature map, inevitably involving irrelevant or redundant interactions, which may introduce noise and impair model performance. To address this issue, we incorporate a squared ReLU-based sparse self-attention (SSA) mechanism that filters out features with low query–key matching scores, thereby suppressing the negative impact of weak correlations [23]. Nonetheless, due to the potential over-sparsity of SSA [24], we further integrate a dense self-attention (DSA) branch that employs a softmax function to preserve critical information flow. By adaptively combining the outputs of both branches, our model effectively minimizes redundant or noisy interactions while retaining as much task-relevant information as possible. The overall design of the proposed MASSA block is shown in Fig 1c.

Let $Y_{all}=R^{N\times K\times F}$ denote the features after multi-branch and multi-scale merging, where $K=k_{\text{4}}$, $F=\sum f_{\text{4}}$. Then, we generate the query matrix Q, the key matrix K, and the value matrix V from $Y_{all}$ through linear projection matrices $W_{Q}$, $W_{K}$, and $W_{V}\in R^{K\times D}$ as follows:


$$\begin{array}{c} Q=Y_{all}^{T}W_{Q},K=Y_{all}^{T}W_{K},V=Y_{all}^{T}W_{V} \end{array}$$ (7)

Subsequently, h independent attention heads $Q\text{'},K\text{'},V\text{'}\in R^{N\times h\times F\times d}$ (where d=D//h) are employed to compute attention weights separately. The results from these heads are then merged through linear projection, thereby achieving more comprehensive representations. The attention computation for each independent head can be defined as:


$$\begin{array}{c} A=f(q_{i}^{T}k_{i}/\sqrt{d}+bias)v_{i},i=1,2,...,h \end{array}$$ (8)

Where $q_{i}$, $k_{i}$, and $v_{i}$ represent the i−th attention head’s $Q{\text{'}}_{i}$, $K{\text{'}}_{i}$, and $V{\text{'}}_{i}\in R^{N\times F\times d}$, respectively. d represents the dimension of the features. A denotes the estimated attention, bias represents the learnable relative positional bias, and f(·) is the attention score function.

Most existing studies employ the standard DSA, which computes attention scores for all query-key pairs through a softmax function layer:


$$\begin{array}{c} DSA=SoftMax(q_{i}k_{i}^{T}/\sqrt{d}+bias) \end{array}$$ (9)

Where DSA represents the feature vector after being weighted by dense self-attention mechanism branch, SoftMax(·) denotes softmax function.

Since not all query tokens are closely related to their corresponding tokens in the keys, leveraging the similarity of all generated time-frequency features may result in interactions that are ineffective or redundant for sleep stage detection and may also introduce noise. Intuitively, developing a SSA mechanism to select useful interactions between tokens can enhance feature aggregation, thereby filtering out the negative effects of low query-key matching scores on the aggregated features. To achieve attention sparsity, this study employs a squared ReLU-based function layer (only retaining features with positive values) to eliminate similarities with negative scores and propagate the most useful information flow:


$$\begin{array}{c} SSA=ReLU^{\text{2}}(q_{i}k_{i}^{T}/\sqrt{d}+bias) \end{array}$$ (10)

Where SSA represents the feature vector after being weighted by sparse self-attention mechanism branch,ReLU(·) denotes Relu function.

However, the straightforward application of a ReLU-based SSA may result in information loss and excessive sparsity, causing the learned feature representations to contain insufficient information for subsequent processing. This, in turn, can lead to instability in network training. Therefore, this study equips each branch with adaptive attention scores to fully leverage both paradigms. The attention matrix in the equation can be further updated as follows:


$$\begin{array}{c} A=(w_{\text{1}}*SSA+w_{\text{2}}*DSA)V \end{array}$$ (11)

Where $w_{\text{1}},w_{\text{2}}\in R^{\text{1}}$ are the two normalized weights used for adaptively modulating the dual branches. More specifically, it can be calculated by the following formula:


$$\begin{array}{c} w_{n}=\frac{e^{a_{n}}}{\sum_{i=1}^{N}e^{a_{i}}},n=\left\{1,2\right\} \end{array}$$ (12)

Where $\left\{a_{\text{1}},a_{\text{2}}\right\}$ are learnable parameters, both initialized to 1. This design ensures that sufficient informational features are utilized for subsequent classification operations while filtering out noise interactions from irrelevant time periods.

In general, this dual-branch approach allows the model to benefit from both the efficiency of sparse attention and the comprehensive information capture of dense attention. By adaptively weighing and combining these two types of attention, the model can effectively balance the trade-off between reducing noise and maintaining rich feature representations, thereby improving overall performance and training stability.

### Adaptive cross-modal fusion block

After multi-branch, multi-scale feature extraction and refinement using MASSA, we obtain two sets of high-level representations, $H_{EEG}\in R^{N\times K\times F}$ and $H_{EOG}\in R^{N\times K\times F}$. To fully utilize the complementary information provided by the two modalities, we introduce an adaptive cross-modal fusion module (ACFM), as shown in Fig 1d. The key idea of this module is not simply to concatenate EEG and EOG features, but to learn which modality is more informative at each time step. Specifically, the EEG and EOG representations are first concatenated to form a joint feature representation. This joint representation is then passed through a linear mapping and a softmax operation to generate attention weights, which quantify the relative contribution of each modality. The resulting weights are used to reweight the original EEG and EOG features before fusion. In this way, the module can dynamically emphasize the modality that provides more discriminative information for the current sleep stage, leading to more effective multimodal feature integration.

The module primarily comprises the following steps and components: Firstly, the two sets of high-level representations for the two modalities, learned from the preceding network, are merged through a concatenation operation to form a joint representation H.


$$\begin{array}{c} H=\left[H_{EEG},H_{EOG}\right]\in R^{N\times 2K\times F} \end{array}$$ (13)

Here, [A, B] denotes the concatenation of A and B along a specified dimension.

Subsequently, the concatenated features are passed through a linear mapping layer to compress or re-encode the feature representations, resulting in a mapped feature representation with a dimension of $R^{N\times 2\times F}$. These mapped features are then normalized using a Softmax operation to generate weighting coefficients. These coefficients indicate the relative importance of the two input features at different time steps.


$$\begin{array}{c} Out=Softmax\left(Linear\left(H\right)\right)\in R^{N\times 2\times F} \end{array}$$ (14)

Where $Out\in R^{N\times 2\times F}$ represents the attention weight.

The original features are element-wise multiplied by the weighting coefficients, and the resulting weighted feature representations are merged.


$$\begin{array}{c} \begin{array}{c} Out1,Out2=Split(Out,dim=1)\\ Output=Out1*H_{EEG}+Out2*H_{EOG} \end{array} \end{array}$$ (15)

Where $Out1,Out2\in R^{N\times 1\times F}$ represents the attention weights of the EEG and EOG branches, respectively.

Finally, the weighted feature representations are passed through a fully connected layer for dimensionality reduction and classification.

## Results and discussion

### Datasets and evaluation metrics

#### Datasets.

We evaluated the performance of MMFNet on two benchmark datasets: Sleep-EDF-39 [25] and Sleep-EDF-153 [26]. The Sleep-EDF-39 dataset contains 39 full-night recordings collected from 20 healthy subjects, comprising a total of 42,308 sleep epochs. In contrast, the Sleep-EDF-153 dataset consists of 153 full-night recordings from 78 healthy subjects, with 195,479 epochs in total. Each 30-second epoch was annotated into one of five standard sleep stages—wake (W), N1, N2, N3, and REM—according to the most recent scoring guidelines of the American Academy of Sleep Medicine (AASM) [3].

The de-identified data files used in this study were obtained from a public repository. The authors accessed and downloaded the datasets for the purposes of this research between 14 July 2025 and 17 July 2025. The released files are fully anonymized and do not contain direct identifiers (e.g., names, addresses, or contact information). Therefore, the authors did not have access to any information that could identify individual participants during or after data collection.

This study is a secondary analysis of fully anonymized, publicly available datasets. No new participants were recruited, and no new data were collected by the authors. In accordance with the policies of Aba teachers college, secondary analyses of public de-identified datasets are exempt from additional ethical review. As a result, no separate institutional ethics approval was required for this work.

#### Evaluation metrics.

The performance of the proposed model is comprehensively evaluated using several metrics, including Accuracy, Macro-F1 score (MF1), Cohen’s Kappa (**κ**), Precision (P), and Recall (R). The mathematical formulations are provided in (16)–(21).

Specifically, True Positive (TP) refers to the number of positive samples correctly identified as positive, while False Negative (FN) denotes positive samples incorrectly predicted as negative. False Positive (FP) indicates negative samples that are incorrectly classified as positive, and True Negative (TN) represents negative samples correctly identified as negative. In addition, $p_{o}$ denotes the observed agreement probability, and $p_{e}$ represents the expected agreement probability.

The evaluation metrics are defined as follows:


$$\begin{array}{c} Accuracy\left(ACC\right)=\frac{TP+TN}{TP+TN+FP+FN} \end{array}$$ (16)

$$\begin{array}{c} Precision\left(P\right)=\frac{TP}{TP+FP} \end{array}$$ (17)

$$\begin{array}{c} Recall\;\left(R\right)=\frac{TP}{TP+FN} \end{array}$$ (18)

$$\begin{array}{c} F1-score=\frac{2\times Preci\text{s}ion\times Recall}{Preci\text{s}ion+Recall} \end{array}$$ (19)

$$\begin{array}{c} Macro-F1\left(MF1\right)=\frac{\text{1}}{K}\sum_{i=1}^{K}F{\text{1}}_{i} \end{array}$$ (20)

$$\begin{array}{c} Cohen^{\prime }s\;Kappa=\frac{p_{o}-p_{e}}{1-p_{e}} \end{array}$$ (21)

where *K* denotes the number of classes.

Accuracy (ACC) measures the overall proportion of correctly predicted samples but may be less reliable under imbalanced data distributions. The Macro-F1 score (MF1), calculated as the average of the F1 scores across all classes, mitigates the impact of uneven class distributions and is particularly suitable for multi-class classification tasks. Cohen’s Kappa (**κ**) quantifies the agreement between predicted and true labels while accounting for chance agreement; values closer to 1 indicate stronger consistency. Precision (P) evaluates the proportion of true positives among all predicted positives, reflecting the reliability of positive predictions, whereas Recall (R) measures the model’s ability to correctly detect positive samples. Specificity, in turn, assesses the proportion of true negatives correctly identified, which complements Recall in evaluating classification balance.

To provide a more comprehensive assessment, two evaluation strategies are adopted. First, overall results summarize the model’s performance across the entire test set, offering a macroscopic view of its effectiveness. Second, per-class precision is computed to analyze the model’s behavior on individual classes, thereby highlighting potential disparities across categories. This class-wise evaluation is especially important when handling imbalanced datasets. By combining both overall and class-level analyses, the proposed approach enables a thorough and accurate evaluation of model performance, capturing not only overall trends but also class-specific challenges. In this study, the reported performance metrics are presented as overall results aggregated across all test folds under subject-wise cross-validation, rather than as the arithmetic mean of fold-wise values. This evaluation protocol is commonly adopted in sleep staging studies, because different folds may contain different numbers of test epochs and different class distributions. Therefore, overall metrics computed from all held-out predictions provide a more representative assessment of dataset-level performance.

#### Baselines and experimental setup.

In the experiments of this study, MMFNet was compared with five baselines: SleepPrintNet (2020) [27], AttnSleepNet (2021) [16], MMASleepNet (2022) [28], Cross-modal Transformers (2024) [29], and CareSleepNet (2024) [30].

To ensure a fair comparison, we adopted the publicly available implementations of baseline models and trained them under the same environment and configurations as our proposed network. The Sleep-EDF-39 and Sleep-EDF-153 datasets were evaluated independently, rather than being merged for joint training. For each dataset, single-epoch Fpz-Cz EEG and EOG signals were used as inputs, and all models underwent identical preprocessing and data handling procedures to guarantee consistency. We employed a subject-wise five-fold cross-validation strategy separately on each dataset, where all recordings from the same subject were assigned to the same fold. In Sleep-EDF-39, which contains 20 subjects, one fold containing 4 subjects was used for testing in each round, while the remaining 16 subjects were used for training. The same subject-exclusive evaluation protocol was applied independently to Sleep-EDF-153. Therefore, no subject appeared in both the training and test sets within a dataset, and no overlap or data leakage occurred between the two datasets.

All experiments were implemented in Python 3.8 using the PyTorch 1.12 deep learning framework. Training was conducted on an NVIDIA RTX 4090 GPU. The Adam optimizer was adopted for network optimization, with an initial learning rate of 0.001 and a batch size of 32. To improve reproducibility, the source code for this work has been deposited in an Open Science Framework (OSF) repository and will be publicly released (https://osf.io/tmjbk/overview). The corresponding DOI is 10.17605/OSF.IO/TMJBK.

### Sleep stage classification

We compared MMFNet with five baseline methods on the Sleep-EDF-39 and Sleep-EDF-153 datasets, evaluating the number of parameters (Param), overall accuracy (Acc), precision (P), recall (R), macro F1-score (MF1), and Cohen’s kappa (κ). The results are summarized in Tables 2 and 3.

**Table 2:** The overall performance comparison results of the algorithm proposed in this study with other algorithms on the Sleep-EDF-39 dataset.

Method	Param. (M)	ACC	P	R	MF1	κ
SleepPrintNet	2.0	81.68	71.72	76.52	73.27	72.97
AttnSleepNet	0.5	82.13	72.49	77.60	74.21	73.75
MMASleepNet	0.6	79.18	70.55	**78.33**	71.87	70.02
Cross-modal transformer	0.8	81.42	70.60	76.13	72.50	72.74
CareSleepNet	19.5	80.20	71.19	75.89	72.27	71.14
MMFNet	0.8	**84.26** ^**^	**74.85** ^**^	78.24	**75.91** ^**^	**76.60** ^**^

**Note**: Statistical significance was assessed using subject-level paired t-tests between MMFNet and the baseline with the highest ACC on each dataset. * indicates *p* < 0.05, *** indicates *p* < 0.01, and **** indicates *p* < 0.001.

**Table 3:** The overall performance comparison results of MMFNet on the Sleep-EDF-153.

Method	ACC	P	R	MF1	κ
SleepPrintNet (2020)	79.39	68.81	78.29	71.35	71.14
AttnSleepNet (2021)	79.95	69.88	79.37	72.61	72.04
MMASleepNet (2022)	76.76	66.90	78.15	68.94	68.02
Cross-modal transformer (2024)	79.17	69.50	77.83	71.84	70.97
CareSleepNet (2024)	78.09	67.66	75.01	67.45	69.54
MMFNet	**81.86** ^**^	**71.86** ^***^	77.17	**73.38** ^*^	**74.20** ^***^

Note: Statistical significance was assessed using subject-level paired t-tests between MMFNet and the baseline with the highest ACC on each dataset. * indicates *p* < 0.05, *** indicates *p* < 0.01, and **** indicates *p* < 0.001.

On the Sleep-EDF-39 dataset, MMFNet consistently outperforms competing approaches across most metrics. In particular, compared with the state-of-the-art Cross-Modal Transformer, MMFNet achieves notable improvements of 3.02–3.86% across all evaluation metrics while maintaining a comparable number of parameters. This demonstrates that MMFNet more effectively extracts time–frequency features from EEG and EOG signals than the Cross-Modal Transformer, which is constrained by a fixed multi-scale convolutional kernel size of 50. Moreover, unlike other baseline models that arbitrarily set kernel sizes, our design is guided by signal characteristics, enabling more principled and robust feature extraction.

A similar performance trend is observed on the larger Sleep-EDF-153 dataset, further validating the ability of MMFNet to exploit temporal–frequency information and enhance sleep stage classification performance.

**Fig 2:**
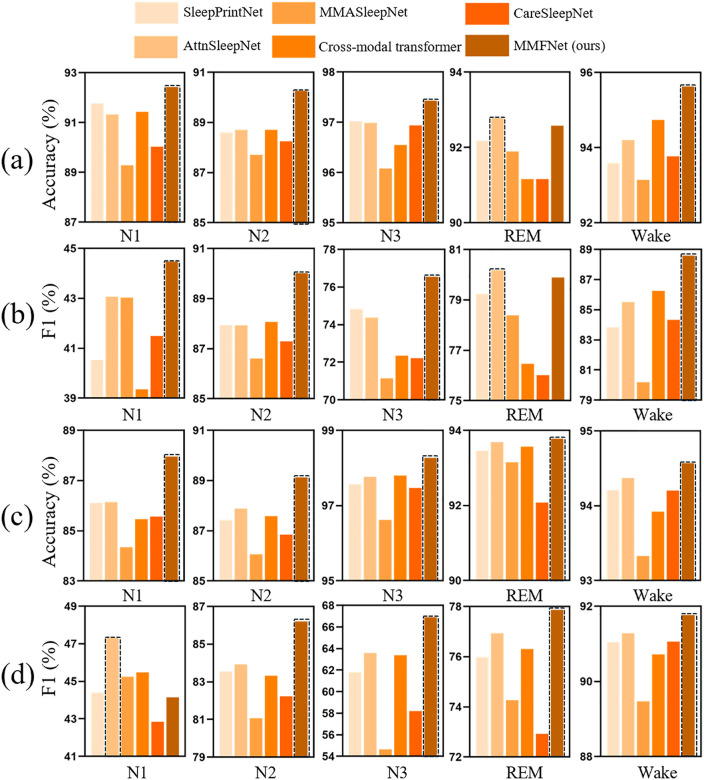
The comparison results of MMFNet with other models in terms of accuracy and F1 score for each sleep stage. (a) and (b) represent the comparison results on Sleep-EDF-39, while (c) and (d) represent the comparison results on Sleep-EDF-153.

Furthermore, Fig 2 illustrates the classification results of the proposed MMFNet and the baseline algorithms for each sleep stage. Our method achieved state-of-the-art performance in nearly all stages. Specifically, on the Sleep-EDF-39 dataset, MMFNet achieved the highest Acc of 97.43% for the N3 stage, which is predominantly characterized by delta waves. This result underscores the effectiveness of our proposed multi-branch time-frequency feature extraction. Additionally, for the REM stage, MMFNet demonstrated comparable performance in both Acc and P relative to other state-of-the-arts algorithms.

**Fig 3:**
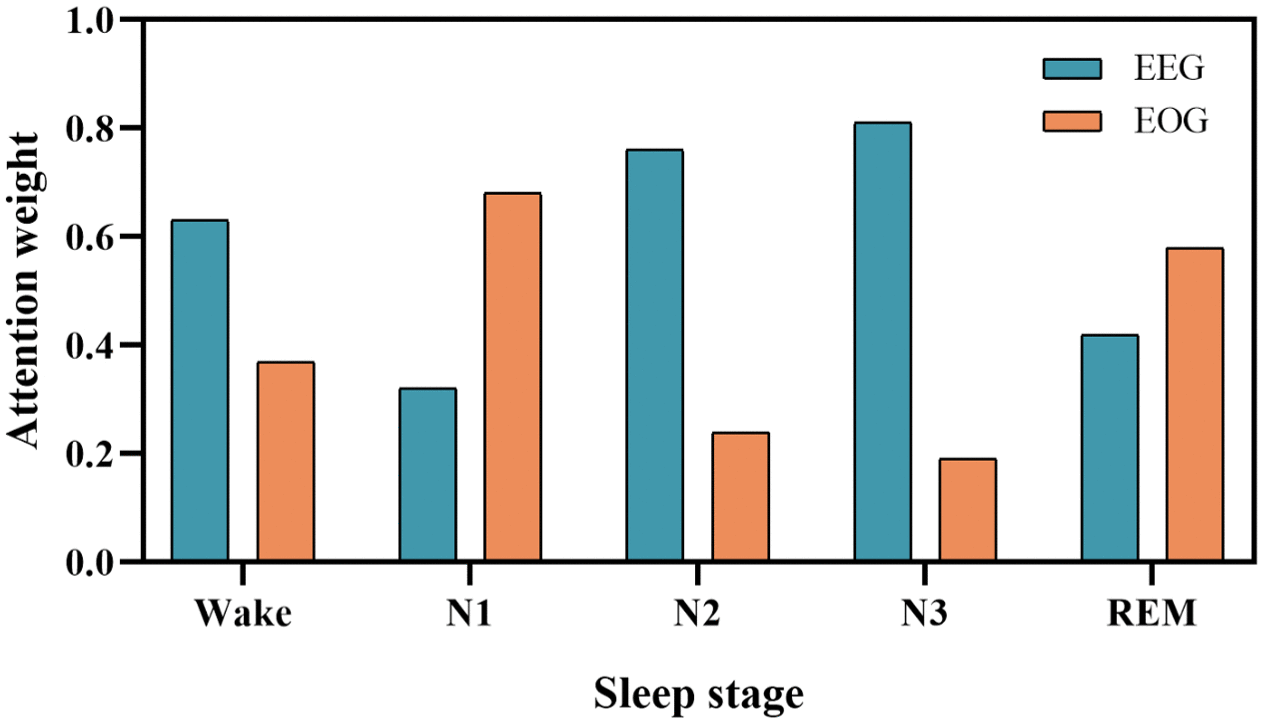
Visualization result of attention weight of ACFM module, in which the attention weight is normalized.

To better elucidate the modality-specific contributions, we visualized the attention weights in the ACFM block (as shown in Fig 3), which provides an intuitive interpretation of the relative importance assigned to EEG and EOG across different sleep stages. The visualization results indicate that EEG signals consistently receive higher attention than EOG during N2 and N3 stages. This observation is physiologically reasonable, as EEG in these stages contains prominent sleep-related waveforms, such as sleep spindles and K-complexes in N2 and high-amplitude delta activity in N3, which play a central role in stage discrimination. Conversely, during N1 and REM stages, EOG obtains greater attention weights than EEG. This pattern can be attributed to the presence of slow eye movements (SEM) that characterize N1 and the distinctive rapid eye movements that dominate REM, both of which serve as critical markers for these stages. Notably, the observed distribution of attention weights aligns well with prior neurophysiological findings [31], thereby confirming the validity of the learned representation and further demonstrating that our model captures stage-specific physiological signatures in a modality-adaptive manner.

**Fig 4:**
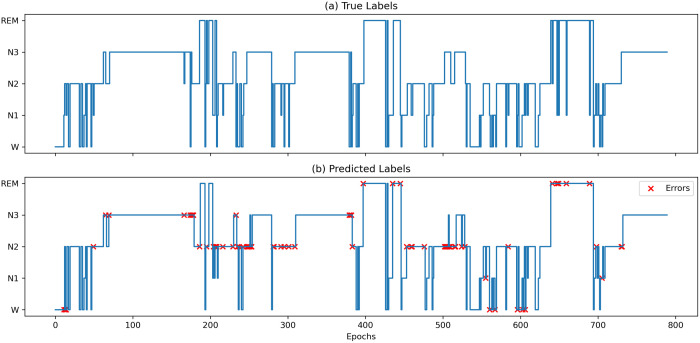
Visualization of sleep stage sequences for the best-performing subject. (a) True hypnogram scored according to AASM guidelines; (b) Predicted hypnogram generated by MMFNet, with red crosses marking misclassified epochs. The vertical axis represents the five standard sleep stages (Wake, N1, N2, N3, and REM), while the horizontal axis denotes the progression of 30-second epochs throughout the night.

**Fig 5:**
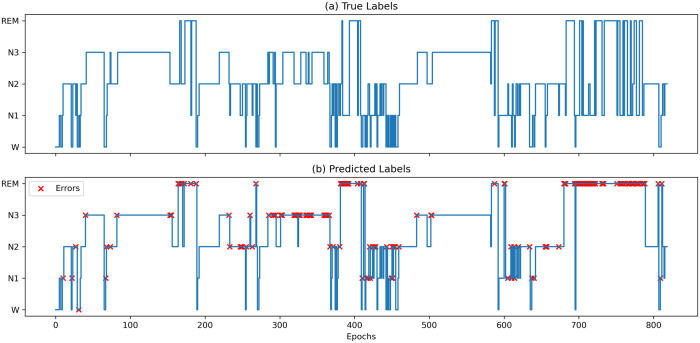
Visualization of the sleep stage sequence for the subject with the lowest classification performance. (a) Ground-truth labels. (b) Predicted sleep stage sequence generated by MMFNet, with red crosses marking misclassified epochs.

Fig 4 and Fig 5 illustrate the classification results of MMFNet for the best- and worst-performing subjects, respectively. As shown in Fig 4, the predicted hypnogram closely follows the ground-truth sequence, with only a small number of errors, mostly concentrated around stage transitions (e.g., N1–N2 and N2–REM). This indicates that the model is capable of accurately capturing both stable sleep stages and their transitions in subjects with clearer physiological characteristics.

By contrast, Fig 5 shows the predictions for the subject with the lowest accuracy. Here, errors are more widely distributed, not only at transitional epochs but also within prolonged segments of N3 and REM, leading to noticeable discrepancies from the true hypnogram. This performance drop highlights the impact of inter-subject variability, signal quality, and individual sleep architecture on classification accuracy. Nevertheless, even in this challenging case, MMFNet still preserves the broad alternation of major sleep stages across the night, reflecting its robustness in capturing macro-level sleep structure.

**Fig 6:**
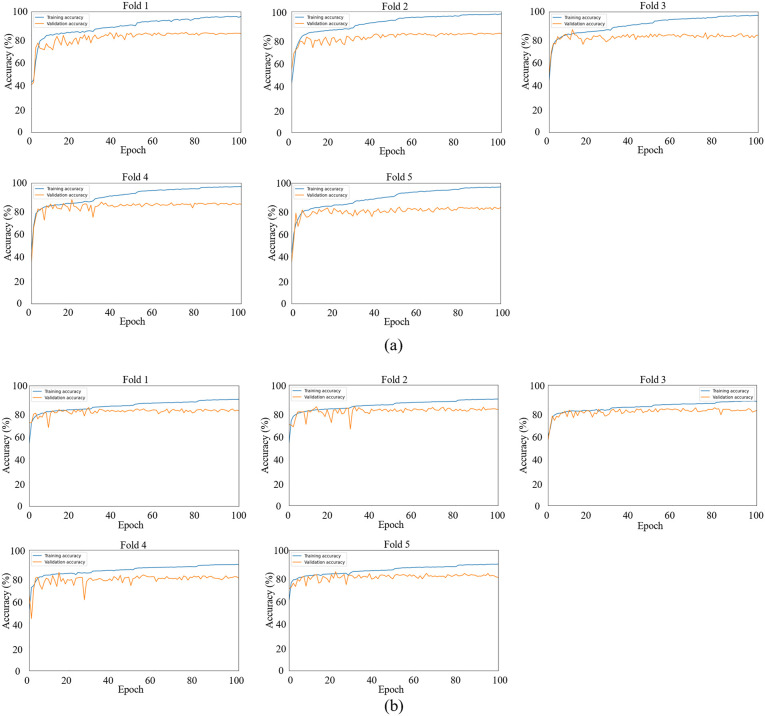
Learning curves of MMFNet under subject-wise five-fold cross-validation on two datasets. Panels (a) and (b) correspond to Sleep-EDF-39 and Sleep-EDF-153, respectively. For each fold, the blue and orange curves denote the training accuracy and validation accuracy across epochs. These curves illustrate the optimization process and generalization behavior of the proposed model on the two benchmark datasets.

The Fig 6 presents the training and validation accuracy curves of MMFNet under five-fold cross-validation on the Sleep-EDF-39 and Sleep-EDF-153 datasets. Overall, the training accuracy in all folds increases steadily with the number of epochs, indicating that the proposed network can be effectively optimized on both datasets. Meanwhile, the validation accuracy also rises rapidly during the early stage of training and then gradually stabilizes, suggesting that the model achieves convergence with reasonable generalization performance. Compared with Sleep-EDF-39, the curves on Sleep-EDF-153 exhibit slightly smaller fluctuations but converge to a comparable accuracy level, which may be attributed to the larger sample size and greater diversity of subjects in the latter dataset. In addition, the gap between the training and validation curves remains relatively controlled across most folds, demonstrating that the proposed model maintains a good balance between representation learning and generalization rather than suffering from severe overfitting. These observations further support the robustness and stability of MMFNet during optimization.

These findings highlight the importance of developing models that not only achieve high average accuracy but also adapt to inter-individual variability. Future work will focus on enhancing cross-subject generalization through domain adaptation and the integration of additional physiological modalities. Ultimately, such efforts will contribute to the realization of personalized sleep health assessment, enabling automated systems to provide individualized insights that can support both clinical diagnosis and daily sleep monitoring.

### Ablation experiment

We conducted a module-based ablation study on the Sleep-EDF-39 dataset to evaluate the contribution of each component within MMFNet and to verify their effectiveness. The ablation settings were as follows:

Base: Only EEG data is processed through the multi-branch time–frequency feature extractor, and the resulting features are directly fused and classified with a fully connected layer.

Base + MASSA Block: On top of the base model, the fused features are further processed using the MASSA block, which adaptively suppresses redundant information and mitigates noise interactions while modeling temporal dependencies.

Base + EOG + MASSA Block: Extending the second variant, an additional EOG branch is introduced. High-level EEG and EOG features are concatenated after the MASSA block and classified through a fully connected layer.

MF-SANet: To conduct an ablation study, the MASSA block in MFFNet was replaced with a standard self-attention mechanism.

MFFNet: Based on the third variant, adaptive fusion of cross-modal features is performed.

**Fig 7:**
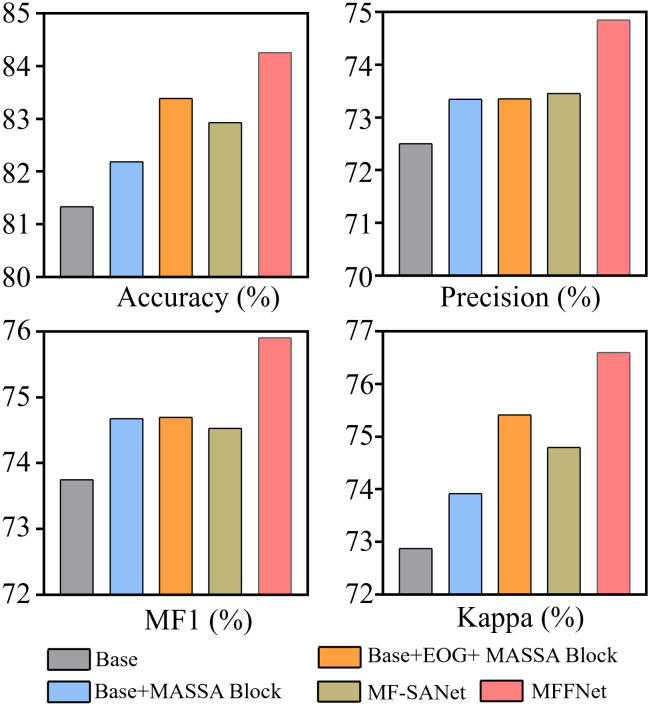
The ablation comparison results of each module proposed in this study.

Based on the ablation study, we can draw the following three conclusions, as illustrated in Fig 7: (1). The inclusion of the MASSA block significantly improves classification performance, confirming the importance of modeling temporal dependencies and reducing redundancy. (2). Comparing the second and third variants demonstrates that the integration of EOG provides substantial complementary benefits, thereby enhancing classification accuracy. (3). The complete MMFNet consistently outperforms all ablated variants across all metrics, showing that adaptive multimodal fusion refines high-level representations and yields superior classification performance. Overall, these findings confirm that each component of MMFNet is not only effective on its own but also works synergistically to enhance sleep stage classification.

### Generalization evaluation on the ISRUC-S1 dataset

To further evaluate the generalization ability of the proposed method beyond the Sleep-EDF dataset family, we additionally conducted experiments on the public ISRUC-S1 dataset [32]. ISRUC-S1 contains overnight PSG recordings from 100 subjects with diagnosed sleep disorders (55 males, aged 20–85 years), and differs substantially from Sleep-EDF in both subject population and acquisition protocol. The original dataset includes six EEG channels, three EMG channels, two EOG channels, and one ECG channel, and is recorded at a sampling rate of 200 Hz. Sleep stages are annotated according to the AASM standard into five categories.

For consistency with our experimental setting, we selected C3-A2 and LOC-A2 as the EEG and EOG channels, respectively, for sleep stage classification. Since ISRUC-S1 was originally recorded at 200 Hz, the selected signals were further downsampled to 100 Hz before being fed into the network. Afterwards, the same epoch-based preprocessing strategy used in the Sleep-EDF experiments was applied to ISRUC-S1, ensuring a consistent input format across datasets and enabling a fairer evaluation of the proposed model under different acquisition conditions.

The comparison results on ISRUC-S1 further confirm the robustness of MMFNet. As shown in Table 4, MMFNet achieved an ACC of 77.45%, P of 76.30%, R of 75.86%, MF1 of 75.81%, and κ of 70.90, outperforming all baseline methods. Compared with the strongest competing baselines, MMFNet improved ACC by approximately 3.1 percentage points, MF1 by 3.46 percentage points, and κ by 3.78 percentage points. These results demonstrate that the proposed architecture generalizes well not only to healthy-subject sleep datasets such as Sleep-EDF, but also to a more challenging clinical dataset with different sampling frequencies, channel configurations, and population characteristics.

**Table 4:** The overall performance comparison results of MMFNet on the ISRUC-S1.

Method	ACC	P	R	MF1	κ
AttnSleepNet (2021)	73.22	70.83	71.95	70.91	65.75
MMASleepNet (2022)	70.75	69.61	70.65	69.08	62.81
Cross-modal transformer (2024)	74.33	72.47	72.39	72.16	66.98
CareSleepNet (2024)	74.32	72.13	73.23	72.35	67.12
MMFNet	**77.45** ^***^	**76.30** ^***^	**75.86** ^***^	**75.81** ^***^	**70.90** ^***^

Note: Statistical significance was assessed using subject-level paired t-tests between MMFNet and the baseline with the highest ACC on each dataset. * indicates *p* < 0.05, *** indicates *p* < 0.01, and **** indicates *p* < 0.001.

### Statistical significance analysis

To further verify whether the performance improvements of the proposed method are statistically meaningful, we conducted subject-level paired t-tests between MMFNet and the strongest baseline in terms of ACC on each dataset. Specifically, the comparisons were performed against AttnSleepNet on Sleep-EDF-39, AttnSleepNet on Sleep-EDF-153, and the Cross-modal Transformer on ISRUC-S1. The significance levels are indicated in Tables 2–4, where *, **, and *** denote *p* < 0.05, *p* < 0.01, and *p* < 0.001, respectively. The results show that MMFNet achieves statistically significant improvements on most of the main evaluation metrics across the three datasets, thereby providing stronger evidence that the observed gains are robust rather than arising from random variation.

## Conclusions

In this study, we proposed MMFNet, an end-to-end framework for automatic sleep stage classification that effectively exploits the time–frequency characteristics of physiological signals. The proposed architecture integrates three key components: (1) a multi-branch, multi-scale feature extraction module, which is designed according to the frequency characteristics of EEG and EOG to capture complementary fine-grained temporal and frequency-aware representations; (2) a Multi-Head Adaptive Sparse Self-Attention (MASSA) block, which models temporal dependencies while suppressing irrelevant and redundant interactions through the adaptive combination of sparse and dense attention branches; and (3) an adaptive cross-modal fusion module, which dynamically integrates high-level EEG and EOG features to enhance the discriminative capability of the learned representations. Extensive comparative and ablation experiments on the Sleep-EDF-39 and Sleep-EDF-153 datasets demonstrated the superior performance of MMFNet and verified the effectiveness of each proposed component. Furthermore, additional evaluation on the ISRUC-S1 dataset further confirmed the robustness and generalization ability of the proposed method under different subject populations and acquisition protocols. In future work, we plan to extend this framework by incorporating additional physiological modalities, such as EMG, respiratory, and cardiovascular signals, and by validating the model on larger and more diverse cohorts. Ultimately, we aim to further advance the clinical applicability of automated sleep staging and contribute to the development of more reliable, scalable, and individualized sleep monitoring systems.
